# Mitochondrial DNA Copy Number in Cleavage Stage Human Embryos—Impact on Infertility Outcome

**DOI:** 10.3390/cimb44010020

**Published:** 2022-01-09

**Authors:** Amira Podolak, Joanna Liss, Jolanta Kiewisz, Sebastian Pukszta, Celina Cybulska, Michal Rychlowski, Aron Lukaszuk, Grzegorz Jakiel, Krzysztof Lukaszuk

**Affiliations:** 1 Invicta Research and Development Center, 81-740 Sopot, Poland; joanna.liss@invicta.pl (J.L.); sebastian.pukszta@invicta.pl (S.P.); celina.cybulska@invicta.pl (C.C.); aron.lukaszuk@invicta.pl (A.L.); grzegorz.jakiel1@o2.pl (G.J.); luka@gumed.edu.pl (K.L.); 2 Department of Medical Biology and Genetics, University of Gdansk, 80-308 Gdansk, Poland; 3 Department of Human Histology and Embryology, Medical Faculty, Collegium Medicum, University of Warmia and Mazury in Olsztyn, 10-082 Olsztyn, Poland; jolanta.kiewisz@uwm.edu.pl; 4 Laboratory of Virus Molecular Biology, Intercollegiate Faculty of Biotechnology of University of Gdansk and Medical University of Gdansk, 80-307 Gdansk, Poland; michal.rychlowski@biotech.ug.edu.pl; 5 The Center of Postgraduate Medical Education, 1st Department of Obstetrics and Gynecology, University of Gdansk, 01-004 Warsaw, Poland; 6 Department of Obstetrics and Gynecology Nursing, Medical University of Gdansk, 80-210 Gdansk, Poland; 7 iYoni App by LifeBite, 10-763 Olsztyn, Poland

**Keywords:** mitochondria, mitochondrial DNA (mtDNA), in vitro fertilization (IVF), fresh embryo transfer, embryo viability, embryo selection, early embryogenesis

## Abstract

A retrospective case control study was undertaken at the molecular biology department of a private center for reproductive medicine in order to determine whether any correlation exists between mitochondrial DNA (mtDNA) content of cleavage-stage preimplantation embryos and their developmental potential. A total of 69 couples underwent IVF treatment (averaged women age: 36.5, SD 4.9) and produced a total of 314 embryos. A single blastomere was biopsied from each embryo at the cleavage stage (day-3 post-fertilization) subjected to low-pass next generation sequencing (NGS), for the purpose of detecting aneuploidy. For each sample, the number of mtDNA reads obtained after analysis using NGS was divided by the number of reads attributable to the nuclear genome. The mtDNA copy number amount was found to be higher in aneuploid embryos than in those that were euploid (mean mtDNA ratio ± SD: 6.3 ± 7.5 versus 7.1 ± 5.8, *p* < 0.004; U Mann–Whitney test), whereas no statistically significant differences in mtDNA content were seen in relation to embryo morphology (6.6 ± 4.8 vs. 8.5 ± 13.6, *p* 0.09), sex (6.6 ± 4.1 vs. 6.2 ± 6.8, *p* 0.16), maternal age (6.9 ± 7.8 vs. 6.7 ± 4.5, *p* 0.14) or its ability to implant (7.4 ± 6.6 vs. 5.1 ± 4.6, *p* 0.18). The mtDNA content cannot serve as a useful biomarker at this point in development. However, further studies investigating both quantitative and qualitative aspects of mtDNA are still required to fully evaluate the relationship between mitochondrial DNA and human reproduction.

## 1. Introduction

The introduction of in vitro fertilization (IVF), more than 40 years ago, was a breakthrough that revolutionized the treatment of infertility and has led to the births of more than eight million children [1]. However, the process of IVF remains inefficient, with only approximately one-third of treatments producing a baby [2]. The success of assisted reproductive treatment (ART) is affected by endometrial receptivity, embryo quality and embryo transfer efficiency [3]. The importance of identification and development of strategies for increasing implantation and pregnancy rates has led to the development of multiple protocols for the analysis of the embryos generated using IVF, with the aim of improving treatment success rates by revealing the embryo(s) most likely to yield a viable pregnancy. Such embryos can then be given priority for transfer to the uterus. Chromosome abnormality is extremely common in human embryos during the first few days of life and is believed to be the single most important cause of implantation failure during IVF treatment [2]. Still, there is a significant percentage of morphologically and chromosomally normal embryos failing to implant which suggests the existence of other factors affecting embryo viability [4,5]. In recent years, several studies have been performed to evaluate mtDNA as a potential biomarker of embryo vitality. However, there is a lot of controversy as studies performed have reached contradicting conclusions [4,6,7,8,9,10,11,12,13,14,15,16,17,18,19,20,21,22,23,24].

Human preimplantation development and embryo implantation involve a range of energetic cellular processes, requiring significant quantities of ATP. Oxydative phosphorylation (OXPHOS), which involves the coordinated action of five complexes, represents the most efficient means of energy generation in the cell. This energy production takes place in mitochondria and depends not only on the nuclear gene expression, but also on transcription of mitochondrial genes. The mitochondrial DNA (mtDNA) is 16.6 kb in length and codes for 13 peptides, which contribute to all complexes required for OXPHOS with the exception of complex 2 [25,26]. The mitochondrial genome resides within the inner membrane of the mitochondrion and it is possible for more than one copy to be present inside each organelle, the number of nucleoids ranging from 1 to 15 per mitochondrion [27,28]. It is possible that the quantity of mtDNA could influence a wide range of vital cellular processes, with important consequences for the function of gametes and embryos.

The way mitochondrial systems have evolved in animals and humans can have profound effects on healthy human reproduction [29]. A number of murine models with targeted deletion of mitochondrial-function genes resulted in infertility or subfertility showing importance of mitochondria in oocyte and early embryo development [30,31,32]. Mitochondria are inherited exclusively from the oocyte and their proliferation is highly regulated in the germline and preimplantation embryo. A sufficient number of mtDNA copies is important for successful fertilization and embryogenesis, since the oocyte mtDNA needs to sustain development until implantation [29,33,34]. Oocytes with an insufficient mtDNA amount fail to fertilize, as inferred from the low mtDNA count in degenerated oocytes [35]. Screening of human preimplantation embryos to select the ones with the greatest developmental potential before transfer is an important goal of reproductive medicine.

The primary end point of this retrospective study is to assess the differences in mtDNA quantity between aneuploid and euploid blastomeres. The secondary points are: the differences in mtDNA quantity between successfully implanted embryos and those that failed to implant; embryos of good and poor morphology; embryos with and without a Y chromosome; embryos from older and younger women. NGS (Next Generation Sequencing) was used to screen for aneuploidy in single blastomeres biopsied from cleavage stage embryos and to evaluate the relative amount of mtDNA (mtDNA/gDNA ratio) in each sample.

## 2. Materials and Methods

### 2.1. Study Design

As most of published studies concerning mtDNA copy number in human embryos were conducted using blastocysts from frozen IVF cycles [7,8,9,10,11,12,13,36], we decided to evaluate the end point parameters by assessing blastomeres biopsied at the cleavage stage that were transferred on day 5 of the same cycle. We aimed to evaluate the significance of the energy production process at such an early stage of embryo development. We have used an approach that allowed for a fresh embryo transfer as the current literature demonstrates mitochondria disfunction and oxidative stress can be caused by cryopreservation [37]. We wanted to exclude the possibility of such an effect in the analyzed embryos. Additionally, in our opinion, such an approach may be reconsidered in the future due to the introduction of rapid and easy sequencing, which does not demand expensive equipment or pooling of many samples. It has been already demonstrated that Nanopore sequencing can be introduced in PGT-A [38]. On the other hand, our previous results [39] showed very high implantation and pregnancy rates for fresh embryo transfer [39].

In this retrospective study, we included infertile couples (*n* = 69; mean female partner age: 36.5 SD 4.9) who underwent IVF with NGS-based preimplantation genetic testing for aneuploidy (PGT-A) of their embryos, obtained in the period from August 2013 until March 2015. At that time, all IVF treatments at the Invicta Fertility Clinics were performed with fresh embryo transfers.

The defined inclusion criteria were (1) indications for preimplantation genetic testing (PGT)—women of reproductive age with 2 or more implantation failures with transfers of TQ embryos, 2 or more miscarriages; age of women more than 35; age of men more than 50; (2) only fresh cycles; (3) patients with a known pregnancy outcome until clinical pregnancy or miscarriage was confirmed. The study excluded patients with intrauterine anomalies. Relevant clinical details, medical history and pedigree were documented during infertility treatment. NGS analysis was carried out at Invicta Medical Laboratories, Molecular Biology Department. The quantity of mtDNA was extrapolated from the NGS data and followed by the analysis of the mtDNA/gDNA ratio.

### 2.2. Stimulation Protocol, Oocyte Retrieval

Anti-Müllerian hormone (AMH) level measurement and transvaginal ultrasonoscan for antral follicle count were performed just before stimulation. Menopausal gonadotrophins were used in monotherapy as described elsewhere [40]. All women were treated with a long agonist protocol starting from oral contraceptives (OCs) (Ovulastan, Adamed, Czosnow, Poland) from the 2nd to the 5th day of the cycle. Triptorelin acetate 0.1 mg (Gonapeptyl, Ferring, Saint-Prex, Switzerland) was administered 14 days after the beginning of the OCs. Fourteen days later (7 days after the end of OC administration), urinary gonadotropins (Menopur, Ferring, Saint-Prex, Switzerland) for ovarian stimulation were administered. Follicular growth was monitored on day 8 using transvaginal ultrasound and assays evaluating serum estradiol (E_2_), progesterone (P) and luteinising hormone (LH) levels until pituitary ovarian down-regulation was reached (i.e., E_2_ concentration < 50 pg/mL). Follicular growth was stimulated by FSH (Menopur, Ferring, Saint-Prex, Switzerland) according to individual endocrine and ovarian ultrasonic response until at least an 18-mm-diameter dominant follicle was observed.

Oocyte pick-up was performed 36 h after the administration of 5000 IU of hCG (Choragon, Ferring, Saint-Prex, Switzerland). The cumulus–oocyte complexes were isolated into Flushing Media (Orgio, Medicult Media, Denmark) under standard IVF condition. Eighteen hours after ICSI procedure, oocytes were assessed for fertilization.

### 2.3. Embryo Culture, Biopsy and Transfer

Embryos were cultured in G-series media (Vitrolife, Västra Frölunda, Sweden) in low oxygen atmosphere. Embryos were classified according to the scale of Cummins et al. [34]: good—6–8 cells, A-B class (equal size and fragmentation of blastomers 0–10%), poor—6–8 cells, C class (differences in blastomers size, fragmentation 11–25%). Only embryos graded as A or B were included in the present study.

Biopsies were performed on day-3 post fertilization when the embryos reached the 6–8 cells stage by selecting a single blastomere that underwent three mitotic divisions. Laser technology (telecomunnication’s laser Anritsu 1488 nm in Saturn 3 RI) was used to create an opening in the zona pellucida that encapsulates each embryo. Single blastomere was gently aspirated. After the biopsy each embryo was washed, transferred to G2 medium (Vitrolife, Västra Frölunda, Sweden) and cultured for two more days. In each case embryos were transferred to the uterus on day-5 during the same cycle (fresh transfer).

### 2.4. Cell Lysis and Whole Genome Amplification

Cell lysis and whole genome amplification (WGA) of biopsied samples was performed using the PicoPLEX Single Cell WGA Kit (New England BioLabs Inc., Ipswich, MA, USA) according to the manufacturer’s protocol.

### 2.5. Next-Generation Sequencing (NGS)

Concentration of DNA after WGA was quantified with Qubit 2.0 Fluorometer and Qubit dsDNA HS Assay Kit (Invitrogen, Waltham, MA, USA).

An Ion Xpress Plus Fragment Library Kit (Ion Torrent, Waltham, MA, USA) was used for library preparation according to the manufacturer’s protocol. Barcoded libraries (Ion Xpress Barcode Adapters kits (Ion Torrent, Waltham, MA, USA)) were clonally amplified with The Ion PGM™ Template OT2 200 Kit (Ion Torrent, USA) using Ion One Touch 2 System.

All samples were diluted to a concentration of 24 pmol and pooled prior to clonal amplification. After chip loading, sequencing was performed using Ion PGM™ Sequencing 200 Kit v2 (Ion Torrent, Waltham, MA, USA) on Ion 314 and 316 chips. Up to 32 samples were barcoded using and multiplexed together and sequenced on the same chip. Preliminary analysis, e.g., base calling and read mapping against the human genome reference sequence (hg19) were performed with Ion Torrent Suite Software (Ion Torrent, Waltham, MA, USA).

Data were analyzed using the Coverage Analysis (V. 5.12.0.0) plugin in the Torrent Suite V. 5.12.3 (Life Technologies), providing the percentage of DNA sequence reads mapped to each chromosome. Read coverage for each chromosome was corrected for GC-bias and aneuploidy detection was performed with reference to results previously obtained from 75 male and 73 female samples, processed in order to establish a baseline for euploid samples. The percentage of the reads derived from a given chromosome for an embryo sample was divided by the reference value for the same chromosome as described by Lukaszuk et al. [39]. Chromosomal gains were associated with ratios >1.5 and losses with ratios <0.5. No mosaicism was reported or considered for calculations.

### 2.6. Relative Mitochondrial DNA Quantification

For each sample, the number of mtDNA reads obtained after analysis using NGS (carried out as described above) was divided by the number of reads attributable to the nuclear genome as described by Wells et al. [41]. This provided a relative quantification of the amount of mtDNA in each sample. For aneuploid embryos, figures were adjusted to take into account loss or gain of nuclear DNA reads attributable to the presence of aneuploidy, which could otherwise distort the mtDNA quantification results. Finally, in order to make the resulting data easier to read, the small factional values obtained were multiplied by 1000.

### 2.7. Cell Fluorescence Staining for Mitochondrial Presence/Activity

To assess mitochondrial content, MII oocytes and aneuploid embryos from the same patients were stained by MitoTracker Red CMXRos (Invitrogen, USA), which incorporates into active mitochondria. DNA contents were monitored by Hoechst 33342 (Invitrogen, Ipswich, MA, USA) with a standard protocol. The specimens were imaged with a confocal laser scanning microscope (Leica TCS SP8X) with lens 20× oil and analyzed using LAS AF 3.2 software (Leica). Cell fluorescence staining allowed presentation of the localization of mitochondria and nuclei within the oocytes and embryos, and consequently to assess the distribution of mitochondria among the formed blastomeres.

### 2.8. Statistical Tests

To estimate the existing differences in mtDNA quantity the obtained data were analyzed concerning: (1) aneuploid and presence of normal blastomeres; (2) successfully implanted embryos and those that failed to implant; (3) embryos of good and poor morphology; (4) embryos with and without an Y chromosome; (5) embryos from older and younger women.

The data analysis was performed using software system STATISTICA, Version 10 (StatSoft Power Solutions, Inc.). The clinical characteristics and outcomes in the investigated group were compared using the Mann–Whitney U test. Box plots were used for graphical presentation of statistically significant data. A value of *p* < 0.05 was considered statistically significant in all tests.

## 3. Results

### 3.1. Baseline Characteristics

The characteristic of patients and clinical details are provided in Table 1.

In the study group, 27 pregnancies were obtained, confirmed by at least one fetal sac and heartbeat (65.5% clinical pregnancy rate per embryo transfer). Four (14.8%) of them were miscarried between 7 and 12 weeks of pregnancy. Twenty-three pregnancies were carried to term resulting in the birth of 33 healthy babies.

### 3.2. mtDNA Quantification

**Figure 1:**
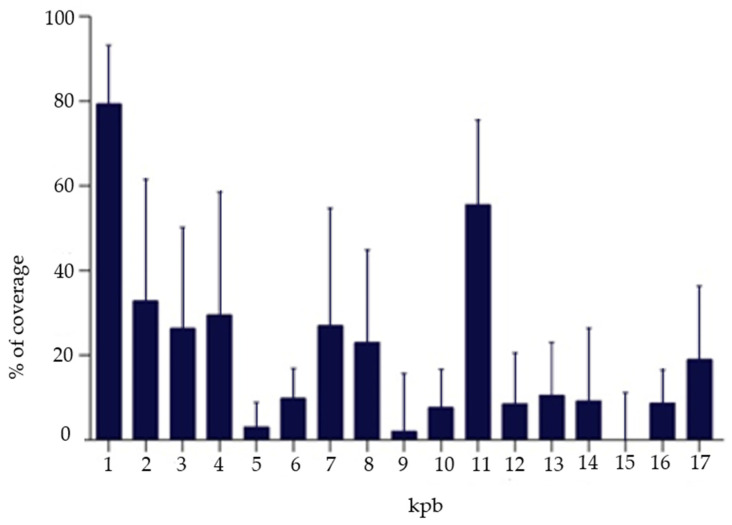
Percentage coverage for mitochondrial sequence after next generation sequencing performed on DNA from single cell after WGA.

Conducted NGS analysis showed that, on average, ~0.7% of mapped reads were mitochondrial. Interestingly, in all cases two regions of the mtDNA were preferentially amplified—from nucleotide position 65 to 500 and from 9950 to 10270 (Figure 1). From region 65 to 500, nucleotide position corresponds to the non-coding area called the D-loop or control region [42]. This cluster contains promotors for the transcription of both heavy and light strands of mtDNA playing a crucial role in transcription and replication of mtDNA [43,44]. Mutations in the D-loop are reported to influence repeated pregnancy loss [45]. Region from 9950 to 10270 nucleotide position covers COX3, TRNG and ND3 genes encoding as follows 3 cytochrome c oxidase, tRNA glycine and NADH dehydrogenase 3 [42].

The mtDNA/gDNA ratios from 314 embryos obtained in the 69 cycles of IVF treatment were analyzed. Interestingly, in some cases results received from embryos from the same patient showed extreme differences.

### 3.3. Ploidy Status

**Figure 2:**
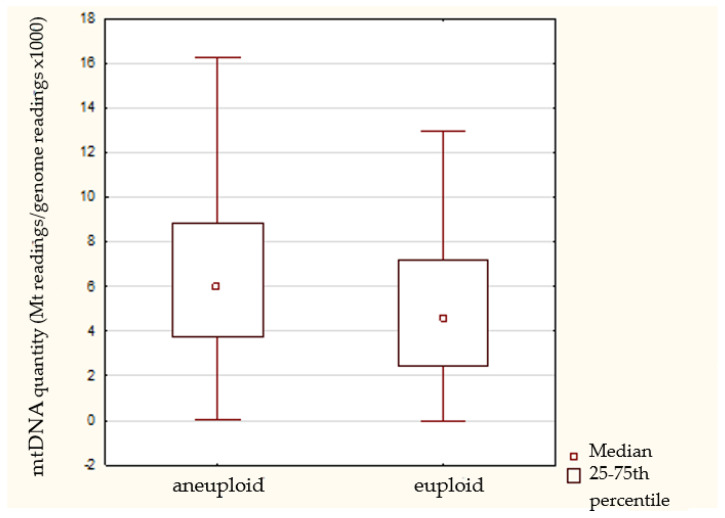
Boxplot (median, hinges: first and third quartiles) showing mtDNA/gDNA ratios obtained for aneuploid and euploid groups of analyzed embryos. NGS analysis of 293 blastomeres showed a statistically significant increase (*p* < 0.004) in the mtDNA levels occurring in the presence of aneuploidy status.

For 93.3% (*n* = 293) of 314 total biopsied embryos, we obtained NGS results. Among the 293 embryos, 89 were euploid and 204 were aneuploid. Comparison of the relative amount of reads between samples according to ploidy status showed that aneuploid embryos tended to have greater quantities of mtDNA (*p* < 0.004; Mann–Whitney U test) (Figure 2, Table 2).

### 3.4. Embryo Quality

In the entire group of 293 embryos 261 had good morphology (A–B class in the scale of Cummins) and 32 had poor morphology (C class in the scale of Cummins). The mtDNA/gDNA ratios were not found to differ significantly between analyzed groups (Table 2). Additionally, the relationship between embryo quality and relevant mtDNA amounts was evaluated for euploid embryos (transferred or cryopreserved for future use); however, the difference was statistically insignificant (Table 2).

### 3.5. Embryo’s Genetic Sex

The distribution of the mtDNA quantities was also evaluated for embryos with and without chromosome Y; however, we did not find any relationship between the mtDNA/gDNA ratios and embryo’s genetic sex neither for all the analyzed embryos or for only the euploid ones (Table 2).

### 3.6. Maternal Age

The relationship between mtDNA relative amounts and female age at the time of the treatment was also considered. The mean female age was 36.5 years (SD 4.9). The mtDNA/gDNA ratios were calculated for two age groups (<37 and ≥37 years of age) to determine the distribution of the mtDNA amounts by female age. No significant difference was observed within analyzed groups (Table 2).

### 3.7. Embryo Transfer Outcomes

**Figure 3:**
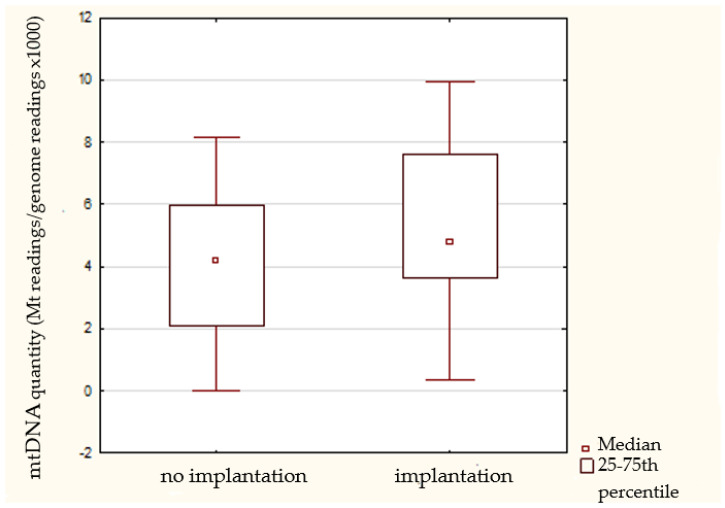
Boxplot (median, hinges: first and third quartiles) showing mtDNA/gDNA ratios obtained for implanted and non-implanted groups of transferred embryos. NGS analysis of 62 transferred embryos showed no significant difference in the quantity of mtDNA between implanted and non-implanted embryos.

From among the 89 normal embryos, 62 were transferred and 27 were frozen. The implantation potential of 62 transferred euploid embryos was considered in relation to mtDNA content of the corresponding biopsied cell. In 22 cases, a single embryo was transferred, while in the remainder two embryos were used. No significant difference was seen in the quantity of mtDNA in embryos that implanted compared with those that failed to implant (Figure 3, Table 2). Nevertheless, we noted a trend toward a decreased mtDNA quantity in non-implanted embryos.

### 3.8. Mitochondrial Presence

**Figure 4:**
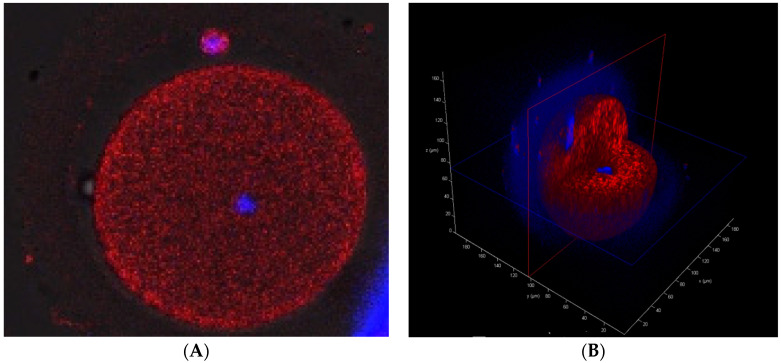
(**A**)—the MitoTracker Red CMXRos (Invitrogen, Ipswich, MA, USA)-stained MII oocyte. Mitochondria are stained red, nuclei are stained blue. (**B**)—the oocyte in the 3D cross-section.

**Figure 5:**
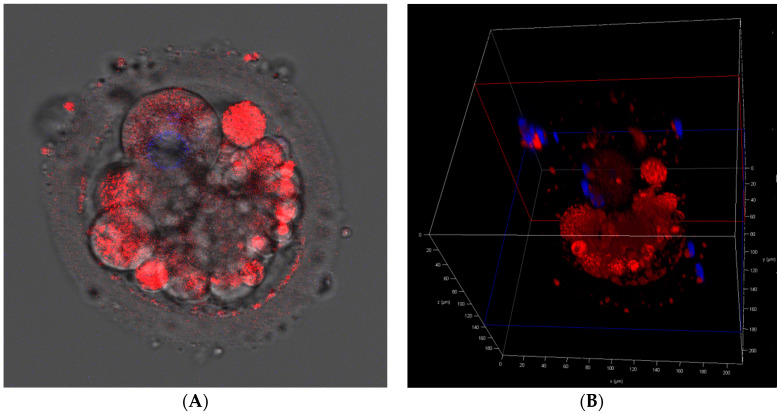
(**A**)—the MitoTracker Red CMXRos (Invitrogen, Ipswich, MA, USA)-stained day-3 embryo. Mitochondria are stained red, nuclei are stained blue. (**B**)—the embryo in the 3D cross-section.

Localization of mitochondria in MII oocytes (Figure 4) and day-3 aneuploid embryos was compared (Figure 5). The comparison confirmed that after fertilization the oocyte’s mitochondria are segregated asymmetrically among the formed blastomeres [46,47]. It is thought that the reduction in mtDNA copy number continues until blastocyst formation [48].

## 4. Discussion

Aneuploidy is the principal reason why the majority of human preimplantation embryos fail to establish a viable pregnancy [2]. Nonetheless, it is clear that even the transfer of euploid embryos to the uterus during IVF treatment cycles does not guarantee the successful initiation of a healthy pregnancy. Some implantation failures may be related to inadequate receptivity of the endometrium or immunological factors [3,49]. However, in most patients such maternal factors are likely to be of secondary importance when compared with other embryonic problems, yet to be defined.

Modern methods for PGT (preimplantation genetic testing) provide highly accurate detection of aneuploidy in cells removed from human preimplantation embryos, allowing the identification of euploid embryos. NGS offers the best chance of fulfilling the need for in-depth analysis of cellular function and health, revealing the embryos that should be prioritized for transfer [39]. The use of NGS makes it possible to detect aneuploidy in individual blastomeres with high accuracy, as we and others have demonstrated previously [40,41,50,51,52,53]. A detailed evaluation of the data produced by NGS also permitted the mtDNA content of biopsied cells to be assessed and quantified. Mitochondria are of growing interest in the field of assisted reproduction, however results of performed studies remain controversial.

Mitochondria are involved in multiple vital signaling pathways, metabolism and apoptosis, their crucial role in oocyte and early embryo development has been demonstrated in several studies using murine models [30,31,32]. The mitochondrial turnover during the early stages of embryo development is thought to be very low, and consequently mtDNA levels are expected to be stable for the first few days of life [48].

In this retrospective study, we assessed the differences in mtDNA quantity between: aneuploid and normal blastomeres; successfully implanted embryos and those that failed to implant; embryos of good and poor morphology; embryos with and without a Y chromosome; embryos from older and younger women.

Our results are consistent with the majority of studies, which were performed using cells from the blastocyst stage, showing that there the amount of mtDNA within aneuploid embryos is higher when compared to euploid embryos [7,12,13,17,18,20,21,22,23,24]. We cannot directly base conclusions regarding mitochondrial activity on these results, as it can be an indirect indication of higher energy needs of aneuploid embryos possibly stemming from the initiated repair mechanisms. Contrary results were shown by Bayram et al. [54], who stated that euploid and aneuploid embryos with equal number of blastomeres contain the same number of mtDNA copies per cell. However, the interpretation of their results is hampered by the lack of a description of the WGA protocol used in the study. There are several WGA methods which differently influence the amplification of mtDNA in comparison to gDNA. Moreover, the sample size is too small to obtain a statistically significant result, even for 33% difference for five-cell embryos. On the other hand, the presented MitoTracker staining (Figure 5) denied the presence of the symmetrical distribution of mitochondria among formed blastomeres. If aneuploid embryos were dividing at a slower rate and the decrease in number of mitochondria was a linear function of cell division, blastomeres biopsied after two mitotic division would present a two-fold increase in mtDNA content in comparison to cells after third mitotic division. It should have been more than a few or several percent difference as we have presented in this paper. We would also suggest that categorization of embryos into two groups based on the median of the mitoscore leads to oversimplification. In our opinion, clustering would be more appropriate. Probably, the groups would not be equal in number; however, they would be grouped according to their similarity.

Our findings suggest that mtDNA could serve as an additional marker of embryo aneuploidy. However, it seems unlikely that mtDNA measurement could replace the much more definitive diagnoses provided by existing PGT methods. Furthermore, we have presented the asymmetrical distribution of mitochondria among the formed blastomeres (Figure 5). Thus, as the embryo may present heterogeneity of their blastomeres, the obtained results may not be reliable. In some cases of the study, we observed extreme differences of the mtDNA/gDNA ratios for embryos from the same patient which suggests that the choice of a blastomere for biopsy strongly affects the outcomes of mtDNA quantification. In consequence, the use of an arbitrary threshold of mtDNA content as embryo selection criteria must be avoided.

Although significant variation in mtDNA quantity was observed in the embryonic samples assessed, there was no link with embryo morphology, suggesting that it has no effect on early development. However, an impact at later embryonic stages cannot be ruled out as, similarly, the presence of aneuploidy had no detectable effect on morphology at this early stage. Other studies assessing blastocysts from frozen IVF cycles also demonstrated no correlation between these two factors [9,12]. On the contrary, Wang et al. [13] showed decreased mtDNA content in higher quality trophoblasts.

Importantly, we also explored the possibility of a link between relative mtDNA quantity and a chromosomally normal embryo’s ability to implant in the uterus. The study of Fragouli et al. [21] has indicated that elevated quantities of mtDNA are associated with implantation failure, and suggested that there is a threshold of mtDNA above which no embryo is able to implant. Similar findings showing that a high mtDNA copy number in euploid embryos correlates with a lower embryo viability and implantation rate for both blastocysts and blastomeres was also demonstrated by several investigators in subsequent studies [4,10,12,13]. If confirmed this would represent a revolutionary discovery, significantly improving the identification of viable embryos, and consequently boosting the success rates of fertility treatments. Unfortunately, the current study suggests that, at the cleavage stage (three days post-insemination), the quantity of mtDNA does not provide useful information concerning embryo viability. It is not until the blastocyst stage that significant levels of mtDNA replication begin to occur, so it may not be until this time that expanded mtDNA levels, indicative of compromised implantation potential, are able to manifest. Moreover, no significant difference between mtDNA quantity in embryos that implanted and those that did not was shown by Victor et al. [20], Treff et al. [24] and subsequently by Scott et al. [11]. Similarly, El-Damen et al. [8] showed a trend of lower mtDNA content for blastocysts leading to pregnancy and live birth; however, it was statistically insignificant.

We also evaluated the correlation of mtDNA level with pregnancy outcomes. The obtained results showed no statistically significant difference; however, the sample size was small and such analysis should be performed using a larger study group.

It has been proposed that the reduction in female fertility seen with advancing age may be associated with diminishing functionality of mitochondria within oocytes, perhaps related to an accumulation of defects in the mtDNA caused by many years of free radical exposure. Theoretically, declining mitochondrial capacity could result in decreased aerobic respiration and reduced energy production, leading to poor embryo quality [55]. Tarín et al. [56] have postulated that induction of age-related aneuploidy during oocyte development may involve oxidative stress, and have demonstrated that antioxidant therapy can prevent associated abnormalities in oocyte chromosomal distribution and segregation.

Several studies concerned with the correlation of mtDNA content with maternal age have produced opposite results demonstrating positive, negative or no correlation with maternal age [4,9,11,12,13,17,21,22,23,24,36]. We evaluated results from 314 cleavage stage embryos derived from patients in different age groups, but no correlation between age and mtDNA could be identified. These findings are consistent with majority of studies conducted on trophectoderm biopsies [4,9,11,12,13,17,23]. The positive correlation of female age and mtDNA content was indicated by Fragouli et al. [21,22] and Perez-Sanchez et al. [36]. Fragouli et al. [21] demonstrated statistically significant difference in amount of mtDNA in both, cleavage stage and blastocyst stage of embryos. Although, blastomeres from embryos obtained from younger women were shown to contain higher mtDNA amounts, compared to those from embryos from older women, while in blastocysts the mtDNA amount was shown to increase with female age. Additionally, their subsequent study echoed their findings, yet the observed difference was statistically insignificant [22]. In our opinion, the reliability of these outcomes has to be considered. Contrary findings, the negative correlation of maternal age and mtDNA quantity, were demonstrated by Treff et al. [24]. The study was performed with day-5 and day-6 blastocysts. Embryos biopsied on day 5 were reported to have higher quantities of mtDNA compared with those biopsied on day 6 what might have also affected obtained results.

It has to be highlighted that two regions of the mtDNA were preferentially amplified by the WGA method used to provide template DNA molecules for our NGS method: from nucleotide position 65 to 500 (HRV2) and from 9950 to 10270. However, this preferential amplification was consistent and reproducible and consequently it did not prevent a reliable assessment of the relative quantity of mtDNA in different samples. However, it does not allow for heteroplasmy assessment. Such analysis demands much deeper sequencing and probably the change of WGA technique which was optimized for aneuploidy testing. Such analysis was performed by Lledo et al. [57], however, their work is difficult to assess in terms of data completeness as it is not known if they succeed in amplification of whole mtDNA sequence and what percentage of heteroplasmy was detected.

Another limitation of our study is that we did not evaluate the pathogenic mtDNA mutations and their implication for reproductive success. Such assessment was conducted by Shamsi et al. [58], although their research was concerned only with mtDNA. Two amplicons (~8 kbp) were amplified and assessed with Sanger sequencing. This method is dedicated solely to mutation analysis. MtDNA level assessment is not designed to assess mutations in mitochondrial DNA just as PGT-A is not designed to assess mutations in the genomic material. This method is designed to assess the number of chromosomes and mtDNA. Their sequences are used only to identify the specific chromosome origin and not to detect any errors. The use of such methods allows for decreased cost and makes the analysis faster, allowing for the assessment of small number of embryos at the same time. Nonetheless, we believe that not only quantitative but also qualitative aspects of mtDNA should be evaluated in subsequent studies.

## 5. Conclusions

In summary, this study confirms that NGS can be used for the simultaneous detection of aneuploidy and measurement of mtDNA content in cells biopsied from human preimplantation embryos. Levels of mtDNA at the cleavage stage have no association with embryo morphology or viability, indicating that this factor cannot serve as a useful biomarker at this point in development and may only be of value when measured at the blastocyst stage [4,21]. However, a relationship between the amount of mtDNA and aneuploidy was observed, suggesting that energetic considerations are important in oocytes and early embryos and might conceivably play a role in the origin of meiotic and/or mitotic aneuploidy. Further studies investigating both quantitative and qualitative aspects of mtDNA are still required to fully evaluate the relationship between mitochondrial DNA and human reproduction.

## Figures and Tables

**Table 1:** Characteristic of patients and clinical details.

Variable	Analyzed Group
No. of subjects	69
Mean (SD) age	36.5 (4.9)
BMI (kg/m^2^)	23.5 (4.3)
No. of subjects with indicated cause of infertility (%)	
Repeated implantation failure	18 (26.1)
Advanced maternal age	19 (27.5)
Male factor	8 (11.6)
Unexplained	22 (31.9)
Endometriosis	2 (2.9)
Mean (SD) duration of infertility (years)	4.3 (3.2)
AMH (ng/mL)	2.42 (1.7)
AFC	13.1 (9.4)
No. of cycles	69
No. of transfers	41
Duration of stimulation—days (SD)	8.6 (1.3)
hMG dose (IU) (SD)	2032.5 (427.5)
No. oocytes retrieved (SD)	7.1 (3.3)
Fertilization rate (%)	75.1
No. of embryos transferred (mean per ET)	62 (1.5)
No. of pregnancies	28
Pregnancy rate per cycle (%)	40.6
Pregnancy rate per ET (%)	68.3
Implantation rate (%)	61.2
Multiple pregnancy rate of pregnancies (%)	11 (39.3)
Ectopic pregnancy (%)	0
OHSS (%)	0
Spontaneous abortion rate (%)	4 (14.3)

**Table 2:** The mtDNA/gDNA ratios for all comparison groups.

Variable	Analysed Group	N (%)	Mean mtDNA/gDNA Ratio (±SD)	Median mtDNA/gDNA Ratio (Quartiles)	U Mann–Whitney *p* Value
Analyzed embryos					
Ploidy status	Euploid	89 (28.3)	6.3 ± 7.5	4.6 (2.8–7.2)	0.004
	Aneuploid	204 (64.9)	7.1 ± 5.8	5.9 (3.7–8.9)	
Embryo morphology (Cummins scale)	A-B class	261 (89.1)	6.6 ± 4.8	5.7 (3.5–8.6)	0.09
	C class	32 (10.9)	8.5 ± 13.6	4.8 (2.4–6.9)	
Sex	With chr. Y	131 (44.7)	6.6 ± 4.1	5.5 (3.8–8.5)	0.16
	Without chr. Y	162 (55.3)	6.2 ± 6.8	5.0 (3.1–7.3)	
Maternal age	<37 years	151 (51.5)	6.9 ± 7.8	5.1 (3.2–8.0)	0.14
	≥37 years	142 (48.5)	6.7 ± 4.5	5.7 (3.8–8.6)	
Euploid embryos					
Embryo morphology (Cummins scale)	A-B class	79 (88.7)	5.6 ± 4.3	4.7 (3.1–7.2)	0.32
	C class	10 (11.3)	11.5 ± 18.9	2.5 (1.5–12.9)	
Sex	With chr. Y	39 (43.8)	6.1 ± 4.4	4.8 (3.6–7.3)	0.22
	Without chr. Y	50 (56.2)	6.3 ± 9.1	4.1 (2.2–7.2)	
Maternal age	<37 years	61 (68.5)	6.2 ± 8.4	4.1 (2.3–7.2)	0.29
	≥37 years	28 (31.5)	6.4 ± 5.2	4.7 (3.2–7.3)	
Transferred euploid embryos					
Implantation status	Implanted	38 (61.3)	7.4 ± 6.6	5.0 (3.7–7.8)	0.18
	Non-implanted	24 (38.7)	5.1 ± 4.6	4.1 (3.3–4.6)	
Pregnancy outcomes	Livebirth	34 (89.5)	7.4 ± 6.5	5.0 (3.7–7.8)	0.22
	Spontaneous abortion	4 (10.5)	7.0 ± 6.9	4.6 (3.9–7.6)	

## Data Availability

The data presented in this study are available on request from the corresponding author.

## Abbreviations

AFC—antral follicle count, AMH—anti-Müllerian hormone, ART—assisted reproductive treatment, ATP—adenosine triphosphate, BMI – body mass index, E2—estradiol, FSH–follicle-stimulating hormone, gDNA—genomic deoxyribonucleic acid, hCG—human chorionic gonadotropin, ICSI—intracytoplasmic sperm injection, IVF—in vitro fertilization, LH—luteinizing hormone, mtDNA—mitochondrial deoxyribonucleic acid, NADH—nicotinamide adenine dinucleotide, NGS—next generation sequencing, OCs,—oral contraceptives, OHHS—ovarian hyperstimulation syndrome, OXPHOS—oxidative phosphorylation, P—progesterone, PGT-A—preimplantation genetic testing for aneuploidies, tRNA—transfer ribonucleic acid, WGA—whole genome amplification.
